# Induction of ventricular fibrillation during both left ventricular and right atrial radiofrequency ablation with a dual energy lattice-tip ablation catheter in patient with implantable cardioverter defibrillator

**DOI:** 10.1007/s10840-026-02382-y

**Published:** 2026-06-13

**Authors:** Heather Wheat, Boldizsar Kovacs, Aman Chugh, Jackson J. Liang

**Affiliations:** 1 Section of Electrophysiology, Division of Cardiology, Department of Internal Medicine, University of Michigan, Ann Arbor, MI USA; 2 Department of Cardiology, Inselspital, Bern University Hospital, University of Bern, Bern, Switzerland; 3 University of Michigan, 1500 E Medical Center Dr, Ann Arbor, MI 48109 USA

Multiple pulsed field ablation (PFA) technologies are now available for use, with expanding applications beyond just atrial ablations and with combined catheters for both PFA and traditional radiofrequency ablation (RFA). Despite its rapid uptake in use, as a community we are continuing to learn more about potential complications of such products and the nuances of each system. One such complication is RFA-induced ventricular fibrillation (VF) when using a dual-energy lattice-tip catheter (Sphere-9, Affera, Medtronic, Minneapolis, MN) in patients with Biotronik (Berlin, Germany) implantable cardiac defibrillators (ICDs).

A 67 year old woman with ischemic cardiomyopathy status post single chamber Biotronik ICD (Acticor 7 VR-T DX, Plexa ProMRI S DX ICD lead) implanted in 2023 presented for combined ablation of frequent premature ventricular complexes (PVCs), atrial fibrillation, and typical atrial flutter. She presented to the laboratory in sinus rhythm with frequent PVCs. A lattice-tip ablation catheter was advanced into the left ventricle after obtaining transseptal access under intracardiac echocardiographic (ICE) guidance. Mapping of her PVC localized to the left ventricular basal inferoseptum with earliest activation – 29 msec pre-QRS, notably overlying known scar observed on her prior cardiac MRI. Initial ablation performed with RFA using atrial settings (5 s lesions at 60% of power output level) at the site of earliest activation (1.5–2 cm away from ICD coil based on fluoroscopy; Fig. 1A and B) immediately resulted in sustained VF requiring external defibrillation (Fig. 1C). Ablation was then continued using PFA without issue. After successful elimination of the PVC, she underwent point-by-point ablation in the left atrium, including uncomplicated pulmonary vein isolation and posterior wall isolation using PFA. The Sphere-9 catheter was then withdrawn into the right atrium for cavotricuspid isthmus (CTI) ablation. The right coronary artery was visualized on ICE at the ventricular end of the CTI line, and the Sphere-9 catheter was noted on ICE to be 2 cm away from the RV ICD coil (Fig. 1D and E). RFA with the same settings at this site again resulted in sustained VF requiring external defibrillation (Fig. 1F). Subsequent lesions away from the annulus were performed using PFA with no further induction of VF and resulted in bidirectional block across the CTI. The patient remained stable for the remainder of the procedure and device interrogation both immediately post-procedure and the following day prior to discharge demonstrated stable lead and generator parameters with no evidence of device malfunction.

**Fig. 1:**
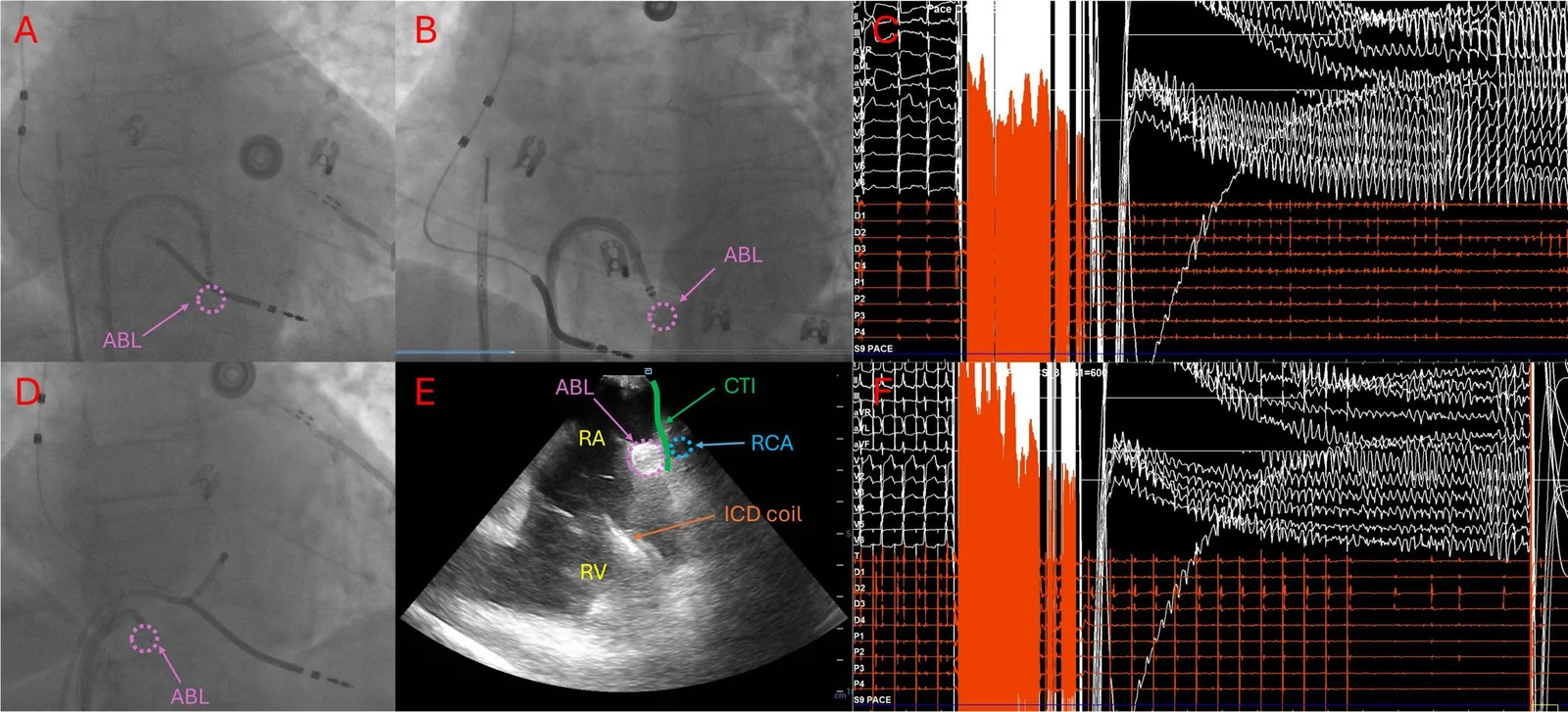
Panels **A** (RAO view) and **B** (LAO view) shows fluoroscopic position of the ablation catheter (ABL) in relation to the ICD coil during ablation from the left ventricular basal inferoseptum. Panel **C** shows induction of sustained VF during RFA targeting PVC from left ventricular basal inferoseptum requiring external defibrillation. Panel **D** (RAO view) shows fluoroscopic position of the ablation catheter (ABL) in relation to the ICD coil during ablation at the ventricular end of the cavotricuspid isthmus. Panel **E** shows intracardiac ultrasound image (right/left inverted) showing location of ablation catheter (ABL) in relation to RV ICD coil during RFA at ventricular end of cavotricuspid isthmus directly on the right coronary artery (RCA). Panel F shows induction of sustained VF during RFA at the ventricular end of the cavotricuspid isthmus requiring external defibrillation

Inadvertent VF during RFA using a lattice-tip dual energy catheter in proximity to an RV defibrillation coil in patients with Biotronik ICDs and RV leads has been previously described from the right atrium, right ventricle and left ventricle [1–3]. The proposed mechanism for such phenomenon includes shunting of current delivery from the RF catheter to the ICD can, transformation of RF to a 40–45 Hz frequency and conduction of this to the myocardium resulting in VF induction. In our case, the ablation catheter appeared to be $\:\ge\:$ 1.5 cm from the defibrillation coil during both LV PVC and CTI ablation. Importantly, ablation at both sites using PFA allowed completion of the procedure. Knowledge of possible interactions with RFA is pertinent when proceeding with catheter ablation with the lattice-tip catheter in order to ensure adequate external defibrillation measures. Caution has to be exercised at sites close to the ICD components to avoid ICD malfunction and follow-up ICD testing is crucial [4].

## Data Availability

No datasets were generated or analysed during the current study.
